# Influence of the DASH Diet on Gestational Weight Gain and Perinatal Outcomes in Women with Pre-Existing Diabetes Mellitus: A Randomized, Single-Blind, Controlled Clinical Trial

**DOI:** 10.3390/life13112191

**Published:** 2023-11-10

**Authors:** Gabriella P. Belfort, Dayana R. Farias, Patricia de C. Padilha, Letícia B. G. da Silva, Karina dos Santos, Mayara S. dos Santos, Lenita Zajdenverg, Elisa Keating, Claudia Saunders

**Affiliations:** 1 Postgraduate Program in Nutrition, Josué de Castro Institute of Nutrition, Federal University of Rio de Janeiro, Carlos Chagas Filho Ave, 373, University City, Rio de Janeiro 21941-590, RJ, Brazil; dayana.farias@nutricao.ufrj.br (D.R.F.); claudiasaunders@nutricao.ufrj.br (C.S.); 2 School of Nutrition, Federal University of the State of Rio de Janeiro, Pasteur Ave, 296, Urca, Rio de Janeiro 22290-240, RJ, Brazil; 3 Maternity School of the Federal University of Rio de Janeiro, Laranjeiras Street, 180, Rio de Janeiro 22240-003, RJ, Brazil; mayarasantos0794@gmail.com (M.S.d.S.); lenitazaj@gmail.com (L.Z.); 4 Unit of Biochemistry, Department of Biomedicine, Faculty of Medicine, University of Porto, Prof. Hernâni Monteiro Ave, 4200-319 Porto, Portugal

**Keywords:** diabetes mellitus, dietary approaches to stop hypertension, fetal macrosomia, gestational weight gain

## Abstract

Background: This study aimed to investigate the influence of the dietary approaches to stop hypertension (DASH) diet on gestational weight gain and perinatal outcomes in pregnant women with pre-existing diabetes mellitus (PDM). Methods: A randomized, single-blind, controlled clinical trial was conducted with 68 pregnant women with PDM throughout prenatal care until delivery (18 weeks) at a public maternity hospital in Rio de Janeiro, Brazil (2016–2020). The standard diet adopted by the control group (standard diet group—SDG) contained 45–55% carbohydrates, 15–20% protein, and 25–30% lipids of the total energy intake. An adapted DASH diet, with a similar macronutrient composition, but with higher calcium, potassium, magnesium, fiber, and reduced saturated fat, was prescribed for the intervention group (DASH diet group—DDG). Student’s *t*- or Mann–Whitney *U* tests were used to compare outcomes between groups. To assess the trajectory of gestational weight gain throughout the intervention between the study groups, linear mixed-effects regression models were used. Results: The DDG had lower gestational weight gain at the fifth (*p* = 0.03) and seventh appointment (*p* = 0.04), with no difference in average total gestational weight gain (SDG: 10 kg [SD = 4]; DDG: 9 kg [SD = 5], *p* = 0.23). There was a trend for a lower length of stay of the newborns (*p* = 0.08) in the DDG without differences for other perinatal outcomes. Conclusions: The DASH diet promoted less variation in gestational weight gain without promoting a difference in total gestational weight gain, and there was no difference between the study groups for perinatal outcomes.

## 1. Introduction

The prevalence of diabetes mellitus (DM) has more than tripled in the last two decades, in association with the growth of obesity, and currently affects about 10.5% of the world’s population. It is estimated that 16.7% of pregnancies are affected by some form of hyperglycemia [1]. Although gestational diabetes mellitus (GDM) is the most common form of diabetes in pregnancy, approximately 10.6% of pregnant women with DM have pre-existing forms already detected before pregnancy, and 9.1% are only diagnosed with pre-existing DM during pregnancy [1].

All forms of hyperglycemia increase the risk of unfavorable perinatal outcomes, such as fetal macrosomia and premature birth, yet pre-existing DM during pregnancy, such as type 1 and type 2 diabetes mellitus (T1DM and T2DM, respectively), is associated with even greater risks [1,2,3]. Other outcomes associated with decompensated DM (hyperglycemia) are congenital malformation, miscarriage, fetal death, prematurity, neonatal hypoglycemia, and respiratory distress, as well as an increased likelihood of developing obesity and T2DM in adult life [3,4].

The American Diabetes Association (ADA) [3] recommends individualized nutritional care for all individuals with DM to help the achievement of adequate glycemic control. Nutritional care is particularly important during pregnancy to prevent postprandial hyperglycemia and nocturnal hypoglycemia, which can impair fetal development [3,5]. Given the above, some researchers have investigated the effect of eating habits on glycemic control and perinatal outcomes and confirmed that diet in association with drug therapy has a positive influence [6,7].

The dietary approaches to stop hypertension (DASH) diet is one of the dietary therapies that seem to reduce the risk of cesarean delivery, lower weight, and ponderal index at birth, as demonstrated by Asemi et al. [8] and Yao et al. [9], who investigated the effect of this diet on women with GDM. In addition to the beneficial effects of the DASH diet for the fetus, the adoption of this diet by women with GDM can also contribute to improving maternal health by reducing fasting and postprandial blood glucose, serum lipids, and maternal blood pressure [10].

In addition to the effect on maternal glycemia, nutritional interventions also seem to assist in controlling weight gain during pregnancy, which is also a factor that increases the risk of fetal macrosomia [7,11,12]. In a randomized controlled clinical trial with healthy American pregnant women, Van Horn et al. [13] observed that the DASH diet brought about lower gestational weight gain than a control diet.

However, to date, no study has investigated the influence of the DASH diet on the perinatal outcomes of pregnant women with pre-existing DM. The present study, therefore, aims to investigate the influence of the DASH diet on the gestational weight gain and perinatal outcomes of pregnant women with pre-existing DM.

## 2. Materials and Methods

### 2.1. Study Design

The present study is derived from the DASDIA research (DASh diet for pregnant women with DIAbetes), which was a randomized, controlled, single-blind clinical trial with two treatment arms. The study protocol was registered on the Brazilian Clinical Trials Registry platform (Rebec—RBR-4tbgv6). The trial was conducted between November 2016 and March 2020 at a public maternity hospital (Maternidade Escola da Universidade do Rio de Janeiro, ME-UFRJ, Rio de Janeiro, Brazil), which is a reference for the prenatal care of women with DM in the municipality of Rio de Janeiro, Brazil. Pregnant women who received prenatal care at the aforementioned study site were referred via the Regulation Center of the Municipality of Rio de Janeiro (SISREG) or by a professional who performed prenatal appointments and identified a need.

**Figure 1:**
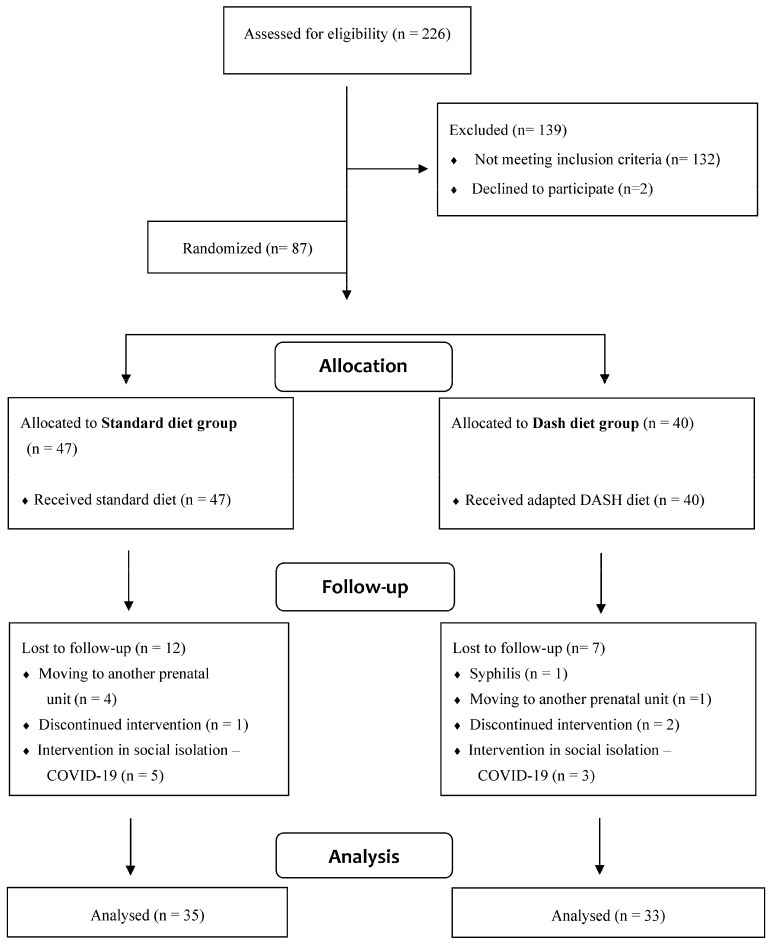
Summary of patient flow.

Two hundred and twenty-six pregnant women were invited to participate in the study by a nutritionist researcher, and 87 pregnant women who accepted and met the inclusion criteria were randomized to the following study groups: the standard diet group (SDG), which represented the control group, or the DASH diet group, which represented the intervention group (DDG, Figure 1).

### 2.2. Participants

All participants received prenatal care at the study site and met the following inclusion criteria: chronological age ≥18 years at conception, diagnosis of DM (types 1 or 2) with onset before pregnancy, single fetus, gestational age ≤28 weeks at recruitment, nonsmoker, and nondrinker. If a candidate had chronic systemic arterial hypertension, they were still included, provided it was mild and controlled (systolic blood pressure ≥140 and <160 mmHg and/or diastolic blood pressure ≥90 and ≤110 mmHg) and they were not diagnosed with hypertensive disorders of pregnancy (HDP—gestational hypertension, pre-eclampsia, or eclampsia). Women with treated and controlled hypothyroidism were also included. The study excluded women with DM complications, such as nephropathy, retinopathy, heart or liver diseases, or other chronic or infectious diseases that could alter the course of pregnancy, and those who had eating disorders.

All pregnant women received prenatal care from a multidisciplinary team throughout pregnancy. Data were collected by trained nutritionists by consulting the women’s (prenatal and postpartum) and newborns’ medical records and by interviews with the participants during the appointments.

### 2.3. Randomization

The participants were randomly allocated to the SDG or DDG by generating a list of random numbers using Microsoft Office Excel^®^ 2016 (Microsoft Corporation). Whenever a new participant was enrolled, they were sequentially allocated a number from this list of random numbers by nutritionist researchers. Women who, when entering the study, received an odd number on the list were allocated to the SDG. Pregnant women who received an even number were allocated to the DDG.

### 2.4. Nutritional Intervention

Both groups received nutritional care throughout their pregnancy, which provided for a minimum of six individual appointments with the nutritionist, scheduled for the same day as the prenatal consultation with the multidisciplinary team. At all the appointments, the women received individualized guidance and, to improve their adherence to the diets, pregnant women who belonged to the DDG received a portion of seeds (200 g), nuts (150 g), and skimmed milk powder (280 g) on each visit, while pregnant women from the SDG received a portion of oats (250 g) and a portion of low-fat milk powder (1–2%, 300 g). At their first consultation, all the women received one bottle (500 mL) of extra-virgin olive oil.

The women from both groups who had low calcium intake (<900 mg/day) were prescribed a daily supplementation of 1 g of calcium carbonate (500 mg of elemental calcium) from the 20th gestational week [14].

#### Standard Diet and Adapted DASH Diet

The standard diet prescribed was the one that was already advised in prenatal nutritional care for pregnant women with pre-existing diabetes at the maternity unit where the study took place, and it followed the guidelines of the American Diabetes Association [3,15].

The DASH diet adopted was the adapted DASH diet for the population of Brazilian pregnant women with pre-existing DM [16]. This study presents secondary results from the randomized, controlled, single-blind clinical trial. Details on the diets applied in the intervention for DDG and SDG are described in the publications by Belfort et al. [17] and Fagherazzi et al. [18]

### 2.5. Anthropometric Assessment

Current weight (kg) was measured at all appointments using an electronic platform scale (Balmak^®^ - São Paulo, Brazil), and height was measured (m) using a stadiometer attached to the scale. Measurements were taken following the standard protocol adopted at the maternity unit [19]. Pregestational nutritional status was assessed by calculating body mass index (BMI), using the weight measured in two previous months or up to the 13th gestational week. Weekly and total weight gain were then estimated, as recommended by the Institute of Medicine [20]. Weekly and total weight gain were assessed at all appointments with a nutritionist.

Total weight gain (kg) was calculated as the weight gain that occurred from the beginning of the intervention until delivery, using the difference between weight at the first appointment and pre-delivery weight. Estimated total average weight gain per group was used to estimate average weekly weight gain across the intervention period.

### 2.6. Sociodemographic, Clinical, and Obstetric Assessment

The data assessed were marital status (live without a partner/lives with a partner), maternal age at baseline (years), family income, self-reported skin color (white or yellow/black or brown), and level of education (not graduated from high school/graduated from high school or more). Regarding obstetric history, the following were investigated: number of pregnancies (nulliparous/non-nulliparous).

The following clinical data were verified: type of DM, duration of illness (years), glycated hemoglobin (%), presence of chronic systemic arterial hypertension, presence of hypothyroidism, and insulin dose at each appointment. To assess the presence or absence of maternal complications, only complications developed during pregnancy or childbirth were considered (HDP, postpartum hemorrhaging, alteration of amniotic fluid, or gestational hypothyroidism).

In addition, variables related to prenatal and postpartum care were evaluated, such as gestational age at the first prenatal nutritional appointment, total number of nutritional and prenatal appointments, and duration of postpartum hospital stay. Ultrasound was preferably used to evaluate gestational age.

### 2.7. Perinatal Outcomes

For the newborns, the following outcomes were investigated: gestational age, weight, length and cephalic perimeter at birth; presence of macrosomia (birth weight ≥ 4.0 kg) [21] or low birth weight (birth weight < 2500 g) [22]; Apgar score in the 1st and 5th minute of life; type of delivery; newborn’s length of stay in hospital (days); and the following neonatal complications—hypoglycemia and respiratory distress (including cyanosis, tachypnea, and fetal distress).

### 2.8. Variables Studied

The type of diet adopted was the independent variable (standard diet/adapted DASH diet). The outcome variables evaluated comparatively between the groups were total gestational weight gain and weekly gestational weight gain during intervention (kg), assessed continuously; maternal complications (yes/no); maternal hospital stay (days), assessed continuously; birth weight (g), assessed continuously and categorically (macrosomia; low birth weight—yes/no); length (cm) and cephalic perimeter at birth (cm), assessed continuously; gestational age at birth (<37/≥37 weeks); Apgar score in the 1st and 5th minute, assessed continuously; type of delivery (normal/cesarean); hypoglycemia (yes/no); respiratory distress or cyanosis (yes/no); and length of stay of the newborn in the hospital (days), assessed continuously.

### 2.9. Sample Size

The sample size was calculated considering the primary outcome of the DASDIA research. Therefore, a prevalence of HDP was estimated at 25%, and a type 1 error of 5% (α = 0.05), a test power of 80%, and an effect size w = 0.5 were considered [23]. It was estimated that each study group should have a minimum of 16 participants [10], but when considering the possibility of a 20% loss throughout the study, a final sample number of 20 participants for each group was obtained.

### 2.10. Statistical Analysis

The sample was described using measures of mean and standard deviation [SD] or median and interquartile range [IQR]) and relative and absolute proportion. The evaluation of data distribution was performed using the Shapiro–Wilk’s test. Student’s *t*-test or the Mann-Whitney U-test was used to compare the continuous variables, according to the normality of the distribution of each variable. The Chi-square test or Fisher’s exact test was applied to compare proportions between study groups.

To evaluate the relationship between the intervention and gestational weight gain across nutritional appointments (represented as study visits), linear mixed-effects regression models were used. Study visits were used as time markers and were included in the model as a random effect. In addition, the model was adjusted for the gestational week of the study visit: intervention time was included as an interaction term in the model in order to assess gestational weight gain, which was different between the groups over time. The dependence of the data was estimated using the unstructured covariance matrix.

Significance was set at 5%, and the analyses were performed using SPSS^®^ Statistics (IBM), version 21.0, and Stata, version 15.0 (Stata Corp., College Station, TX, USA).

## 3. Results

Eighty-seven pregnant women were enrolled and randomly allocated to the DDG (n = 40) or the SDG (n = 47) groups between 2016 and 2020 (until the beginning of the social isolation of the COVID-19 pandemic). There was a 21.8% (n = 19) loss, resulting in 68 women who completed the study (35 in the SDG and 33 in the DDG groups) (Figure 1).

The median age of the study participants was 32 years, and there was no significant difference between study groups. The median gestational age at baseline was similar for the study groups, corresponding to the second gestational trimester, and the mean intervention time for both study groups was 18 weeks (SD = 7) (Table 1).

The study groups had similar sociodemographic and clinical characteristics at baseline. The proportion of women with T1DM was 51% (n = 17) in the DDG and 46% in the SDG (n = 16) (*p* = 0.63) and the median glycated hemoglobin was similar between the groups (SDG: 7 [IQR = 6–8] vs. DDG: 7 [IQR = 6–8]). In both groups, more than half of the women were overweight or obese according to their pregestational BMI (*p* = 0.23). Eighty-three percent of the women in the SDG and 79% in the DDG were partnered, and the majority had graduated from high school or higher education (63% in the SDG and 70% in the DDG, *p* = 0.29) (Table 1).

Regarding diet adherence in both groups, at least 48% of pregnant women showed good adherence to the diet considering all appointments with the nutritionist, and there was no difference between groups at any of the nutritional appointments (*p* > 0.05—Tables in [17]).

No significant difference was observed between the groups for total average weight gain during the intervention (Table 2).

**Figure 2:**
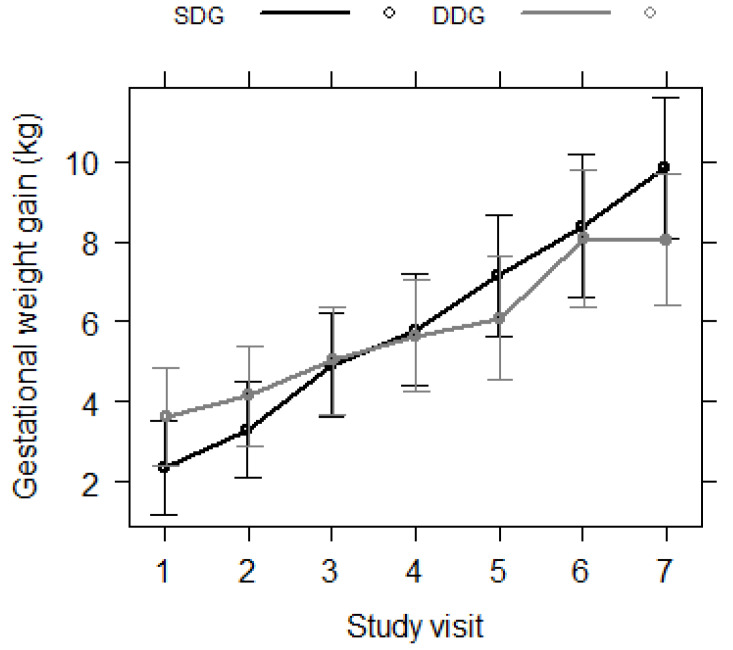
Cumulative gestational weight gain during pregnancy according to intervention group over time (per study visit, which represents nutritional appointments). Linear mixed-effects regression models were used, and the model was adjusted for the gestational week of the consultation.

However, a statistically significant interaction was observed between the intervention group and the study visit. We observed that women in the DDG presented a significantly lower weight gain at the fifth and seventh study visits compared to those in the SDG (Figure 2; Table 3).

Although there were two fetal deaths (one was premature delivery at 34 weeks and 3 days, a newborn with Potter syndrome in the DDG, and the other was born at 36 weeks and 4 days in SDG—of unknown cause), there was no difference in the incidence of maternal complications between the study groups (SDG: 31%, n = 11, v. DDG: 30%, n = 10; *p* = 0.92),the most frequent being pre-eclampsia that affected 18% of women, without difference between study groups (*p* = 0.24; Tables in [17]). There was a mean reduction in glycated hemoglobin for both study groups, with no statistical difference between them (*p* = 0.12—Tables in [17]).

There was no statistically significant difference in the type of delivery, weight, length, cephalic perimeter, gestational age at birth, or other evaluated perinatal outcomes (Table 2). Nevertheless, in the SDG, 29% (n = 10) of the newborns had macrosomia or low birth weight, and in the DDG, 15% (n = 5) had macrosomia or low birth weight. There was a trend towards shorter time to hospital stays for newborns participating in the DDG (3 [IQR = 2,6] vs. SDG: 4 [IQR = 2–9].

## 4. Discussion

In this study, pregnant women in the DDG had less variation in gestational weight gain at the fifth and seventh appointments. However, there were no differences between the groups regarding the type of delivery, prematurity, Apgar score, weight, length, and cephalic perimeter at birth or for neonatal complications (hypoglycemia and respiratory distress). Regarding the prevalence of inadequate birth weight, although no statistically significant differences were observed between groups regarding intervention, the proportion for macrosomia and low birth weight in the DDG tended to be lower.

To date, in the literature, there are no studies that have evaluated the effect of the DASH diet on the perinatal outcomes of women with pre-existing DM. In a study of the effect of the DASH diet on 52 women in Iran with GDM, Asemi et al. [8] observed that there was a lower proportion of cesarean deliveries and that the newborns had smaller head circumference and lower birth weight.

Yao et al. [9] found results similar to those of Asemi et al. [8] when investigating the effect of the DASH diet on Chinese women who developed GDM. They also found a lower incidence of cesarean delivery and lower average weight and length at birth in the group that adopted the DASH diet when compared with the group that adopted the control diet. However, Yao et al. [9] did not detail the differences between the diets tested in terms of food groups.

Some of our results agree with the findings of Jiang et al. [24] in their study of Chinese pregnant women who had chronic arterial hypertension or gestational hypertension, in which the DASH diet was not found to affect the type of delivery or Apgar score. However, they did find that there was a lower proportion of preterm and low birth weight newborns and a greater average length at birth in the group that adhered to the DASH diet.

In a randomized clinical trial developed by Vesco et al. [25], which involved 114 American pregnant women without DM and with obesity, no difference was found between mean birth weight, birth weight z-score according to gestational age, prematurity, hypoglycemia, hyperbilirubinemia, and respiratory distress. However, a significant difference was found for the prevalence of being large for gestational age. As noted in this research, there was also a nonsignificant reduction in the prevalence of macrosomia (14% in the control group vs. 9% in the DASH diet group).

The total prevalence of macrosomia in the present study was 11.8%. These rates are lower than those reported by Owen et al. [26], who evaluated maternal and neonatal outcomes in Irish pregnant women with T1DM and T2DM, who presented a 30% and 20% prevalence of macrosomia, respectively. It was also lower than that observed by Durackova, Kristufkov, and Korbel [27], who found a 54.5% prevalence of macrosomia in pregnant women with T1DM from Bratislava. Siegel et al. [28] also identified a higher prevalence of macrosomia in the newborns of women with DM before pregnancy: 18.8%.

As shown in the studies cited above, the literature is controversial as to which perinatal outcomes are benefited by the adoption of the DASH diet, despite the positive results it seems to bring. In our study, the fact that there were no significant differences for most of the investigated outcomes and there was a lower total prevalence of macrosomia than in the aforementioned research could be due to the multidisciplinary prenatal care that both treatment groups received at the study site, which is specialized in care for DM. All the women received regular, individualized nutritional care, as recommended by the ADA [3], to assist in glycemic control and propose goals of adequate weight gain so that the best pregnancy outcomes could be obtained.

The shorter hospital stay of the newborns in the DDG may be related to the fact that it contained a lower, albeit not statistically significant, proportion of newborns with macrosomia and with low birth weight. Macrosomia is associated with an increased likelihood of hospitalization in intensive therapy because it can predispose to shoulder dystocia, a greater chance of brachial plexus injury, fractures of the clavicle or humerus, and perinatal asphyxia [29,30].

Although there was no difference between the groups for average total gestational weight gain, the DASH diet tended to promote less variation in gestational weight gain since the DDG showed less variation in gestational weight gain between the fourth and fifth and between the sixth and seventh nutrition appointments. This may explain the lower number of newborns who had macrosomia in the DASH group since excessive weight gain during pregnancy of women with DM is associated with an increased risk of macrosomia [12].

Fulay et al. [31], who investigated the influence of adherence to the DASH diet in 1760 healthy American pregnant women, also found no direct relationship between the diet and the outcomes of prematurity and size at birth. However, unlike our study, they found that adherence to the DASH diet favored gestational weight gain for women with pregestational obesity.

Conversely, Van Horn et al. [13] found that the DASH diet had a protective effect on total gestational weight gain when investigating 281 healthy American pregnant women with pregestational overweight or obesity. The average weekly weight gain in the DASH diet group was 0.4 kg, similar to the level observed in our study (median = 0.47 kg/week), which had a high proportion of women with pregestational overweight or obesity.

The research by Vesco et al. [25], previously mentioned, also agrees with our finding about gestational weight gain since they found that the obese women who adopted the DASH diet presented lower average total gestational weight gain than the women who adopted a control diet.

This is the first study we know of that has evaluated the influence of the DASH diet on weight gain and perinatal outcomes in this population of pregnant women. However, a possible limitation of this research is that although the sample size was calculated, the sample obtained does not seem to have been sufficient to prove other benefits of the DASH diet. Furthermore, the referral of pregnant women with DM to the study site is somewhat slow, and this fact interferes with the possibility of starting the intervention in the first trimester of pregnancy in most cases. To obtain a larger sample size, it was not possible to investigate pregnant women separately according to the type of diabetes. This aspect constitutes a limitation of the study since there are metabolic differences between these two types of diabetes. Finally, the influence of the DASH diet was not evaluated in isolation, but only in association with prenatal care.

In conclusion, the adapted DASH diet in conjunction with specialized prenatal care rendered less variation in gestational weight gain but without affecting total gestational weight gain, and it tended to provide a lower length of stay for the newborns in the hospital. Given the relationship between gestational weight gain and negative neonatal outcomes, it is suggested that further research be carried out with this population to reinforce the present findings.

## Figures and Tables

**Table 1:** Characteristics of the pregnant women according to study group (standard diet (SDG) or DASH diet (DDG), Rio de Janeiro, 2016–2020).

Variables	SDG n (%)	DDG n (%)	*p*
**Marital status** (n = 68)			
Lives without a partner	6 (17)	7 (21)	0.67 ^a^
Lives with a partner	29 (83)	26 (79)	
**Family income**			
< Minimum wage	4 (11)	2 (7)	0.68 ^b^
≥ Minimum wage	31 (89)	27 (93)	
**Skin Color** (n = 67)			
White	9 (26)	11 (33)	0.54 ^a^
Brown/Black	25 (74)	22 (67)	
**Education level** (n = 68)			
No complete high school	13 (37)	10 (30)	0.29 ^a^
Complete high school	22 (63)	23 (70)	
**Diabetes, type** (n = 68)			
Type 1	16 (46)	17 (51)	0.63 ^a^
Type 2	19 (54)	16 (49)	
**Parity** (n = 68)			
Nulliparous	12 (34)	12 (36)	0.86 ^a^
Non- nulliparous	23 (66)	21 (64)	
**Pregestational weight status** (n = 68)			
Eutrophic	12 (34)	7 (21)	0.23 ^a^
Overweight or Obesity	23 (66)	26 (79)	
**Presence of chronic arterial hypertensio**n (n = 68)			
Yes			
No	5 (14)	7 (21)	0.45 ^a^
**Presence of hypothyroidism** (n = 68)	30 (86)	26 (79)	
Yes	1 (3.0)	6 (18)	0.05 ^c^
No	34 (97)	27 (82)	
			*p*
**Maternal age, baseline**			
(n = 68, median, years)	32	32	
(IQR)	(26, 35)	(27, 37)	0.86 ^b^
**Gestational age, baseline**			
(n = 63, median, weeks)	14	16	
(IQR)	(11, 20)	(11, 18)	0.39 ^b^
**Diabetes, diagnostic time**			
(n = 68, years, median)	7	9	
(IQR)	(2, 13)	(2, 15)	0.34 ^b^
**Prenatal appointments**			
(n = 67, mean)	12	12	
(SD)	(3)	(4)	0.89 ^d^
**Nutritional appointments**			
(n = 68, median)	6	6	
(IQR)	(5, 6)	(5, 6)	0.76 ^b^
**Glycated hemoglobin, baseline**			
(n = 62, %, median)	7	7	
(IQR)	(6–8)	(6–8)	0.85 ^b^
**Variation in basal insulin dose during the study** (units, n = 67)			
Mean (SD)	33 (30)	29 (30)	0.56 ^d^
**Intervention time, weeks** (n = 68)			
Mean (SD)	18 (7)	18 (7)	0.82 ^d^

SDG, Standard diet group; DDG, DASH diet group; n, sample. ^a^ Chi-square test; ^b^ Mann–Whitney *U* test; ^c^ Fisher’s exact test; ^d^ Student’s *t*-test for independent samples.

**Table 2:** Maternal and perinatal outcomes of pregnant women with pre-existing diabetes mellitus, according to study group (standard diet (SDG) or DASH diet (DDG), Rio de Janeiro, 2016–2020).

Outcomes	SDG			DDG			*p*
	n	Mean	SD	n	Mean	SD	
**Gestational weight gain during intervention** (kg, n = 68)	34	10	4	34	9	5	0.23 ^a^
**Weekly weight gain during intervention** * (kg, n = 68)	31	0.6	0.5–0.8	32	0.5	0.3–0.8	0.22 ^b^
**Maternal hospital stay postpartu**m * (days, n = 66)	34	4	3–8	32	3	2–6	0.15 ^b^
**Birth weight** (g, n = 68)	35	3247	755	33	3335	512	0.58 ^a^
**Length at birth** * (cm, n = 66)	34	48	45–50	32	48	46–50	0.56 ^a^
**Cephalic perimeter** * (cm, n = 64)	32	35	33–36	32	34	33–35	0.59 ^a^
**Apgar, first minute** * (n = 67)	34	8	8–9	33	8	8–9	0.82 ^b^
**Apgar, fifth minute** * (n = 67)	34	9	9–9	33	9	8–9	0.30 ^b^
**Newborn length of stay in the hospital** * (days, n = 66)	33	4	2–9	33	3	2–6	0.08 ^b^
	**n (%)**			**n (%)**			***p***
**Delivery type** (n = 68)							
Vaginal	3 (9)			4 (12)			0.70 ^c^
Cesarean	32 (91)			29 (88)			
**Macrossomia** (n = 68)							
Yes	5 (14)			3 (9)			0.71 ^c^
No	30 (86)			30 (91)			
**Low birth weight** (n = 68)							
Yes	5 (14)			2 (6)			0.43 ^d^
No	30 (86)			31 (94)			
**Gestational age at birth** (n = 68)							
<37 weeks	12 (34)			11 (33)			0.93 ^c^
≥37 weeks	23 (66)			22 (67)			
**Hypoglycemia at birth** (n = 66)							
**Yes**	5 (15)			7 (21)			0.52 ^c^
**No**	28 (85)			26 (79)			
**Respiratory discomfort** (n = 68)							
**Yes**	10 (30)			12 (36)			0.60 ^c^
**No**	23 (70)			21 (64)			
**Maternal complications** (n = 68)							
**Yes**	11 (31)			10 (30)			0.92 ^c^
**No**	24 (69)			23 (70)			

SD, standard deviation; n, sample. * Results expressed in median and interquartile range. ^a^ Independent samples Student’s *t*-test; ^b^ Mann–Whitney U test; ^c^ Chi-square test. ^d^ Fisher’s test.

**Table 3:** Longitudinal association between the intervention group and gestational weight gain during pregnancy (DASH diet and standard diet (SDG) or DASH diet (DDG), Rio de Janeiro, 2016–2020).

	β (95% CI)	*p*-Value
**Intervention**		
DDG	1.3 (−0.1; 3.3)	0.157
**Study Visit**		
2	0.4 (−0.1; 1.0)	0.126
3	1.2 (0.4; 1.9)	0.002
4	1.4 (0.5; 2.4)	0.003
5	2.1 (0.9; 3.3)	<0.001
6	2.5 (0.9; 4)	0.002
7	3.9 (2.4; 5.4)	<0.001
Interaction term		
Study visit 2##DDG	−0.4 (−1.2; 0.4)	0.361
Study visit 3##DDG	−0.8 (−1.7; 0.2)	0.106
Study visit 4##DDG	−0.8 (−1.9; 0.3)	0.153
Study visit 5##DDG	−1.5 (−2.8; −0.1)	0.033
Study visit 6##DDG	−0.3 (−2; 1.4)	0.750
Study visit 7##DDG	−1.7 (−3.3; −0.1)	0.038

Note: The visit during the study, which represents the appointment with a nutritionist, was used as a time marker. The linear mixed-effect regression model was adjusted for gestational age (weeks) at the study visit and was adjusted for gestational age (weeks) at the study visit. *p*-value refers to the maximum likelihood estimator.

## Data Availability

The data presented in this study are available on request from the corresponding author.
